# Evaluating the impact of physical activity cards (PACs) on motor proficiency and enjoyment of physical activities among primary school students in Turkey

**DOI:** 10.1186/s12887-025-06223-1

**Published:** 2025-10-22

**Authors:** Lale Yildiz Cakir, Ali Gurel Goksel, Saadet Sevil Uludag Uyaniker, Aygun Akgul, Kaan Salman, Ayse Demir, Ender Senel

**Affiliations:** 1 Faculty of Sport Sciences, Mugla Sitki Kocman University, Mugla, Turkey; 2 Graduate School of Medical Sciences, Mugla Sitki Kocman University, Mugla, Turkey; 3 School of Physical Education and Sports, Dicle University, Diyarbakir, Turkey

**Keywords:** Physical activity cards, Children, Motor proficiency

## Abstract

This study delved into the potential of the Physical Activity Cards (PAC) program to enhance primary school students’ motor skills and enjoyment of physical activities. The study, conducted in a rural state school, used purposive sampling to select participants. Classroom teacher interviews revealed a need for more PAC usage in physical education and play lessons. Consequently, all third-grade sections from the selected school were included in the study, with 92 students voluntarily participating. Two actively licensed athletes were excluded, leaving a final sample of 49 girls and 43 boys. Using the Test of Gross Motor Development Second Edition (TGMD-2) and the Enjoyment of Physical Activities Scale (PACES), data were collected from 92 students. Results indicated significant improvements in both motor proficiency and enjoyment levels post-intervention. Specifically, the mean scores for motor proficiency increased from 44.77 to 55.02 and for enjoyment, from 37.55 to 47.35. These findings suggest that the PAC program can effectively enhance motor skills and enjoyment of physical activities among primary school students. The study confirmed that PACs improve motor proficiency and affect students’ enjoyment positively. This study underscores the potential of school-based interventions to enhance children’s motor proficiency and enjoyment of physical activities.

## Introduction

In recent years, the world has been increasingly confronted with the urgent risks of a sedentary lifestyle, unhealthy bodies, and mental health issues due to rapidly advancing technology and changing environmental conditions [1]. One of the most significant outcomes of a sedentary lifestyle is obesity, with the highest risk period being childhood between the ages of 6 and 11. According to the Turkey Physical Activity Guide, the average daily duration of sedentary activities such as watching TV, using a computer, internet usage, doing homework, and studying for this age group is six hours [2, 3]. The sedentary lifestyle and intense weight gain observed in this age range can lead to permanent obesity, hormonal disorders, diabetes, hypertension, and cardiovascular diseases [4, 5]. The World Health Organization (WHO) recommends that children aged 5–17 engage in at least 60 min of moderate to vigorous physical activity daily for both physical and mental health. Especially in early childhood, the physical benefits and motor competencies gained through play and physical activities can significantly impact health in later stages of life [6, 7]. At this point, motor competency skills, which do not naturally develop adequately, should be urgently supported through participation in planned physical education and sports activities, especially those involving outdoor play [8].

In Turkey, the educational process in primary, middle, and high schools and equivalent institutions is replete with activities that foster children’s physical and mental development. Lorås [9] underscores that motor development in children is a crucial context that promotes an active lifestyle and that physical education with diverse curricula can enhance overall motor proficiency. Moreover, it is widely acknowledged that physical education and play are practical tools that can influence multiple developmental areas by fostering enjoyment in physical activities [10]. For instance, increased physical activity levels can positively impact children by fostering a love for movement, positive social relationships, and boosting self-confidence [11, 12]. The findings of this study could potentially inform and shape educational and health policies, leading to a more active and healthier future for primary school students in Turkey.

In Turkey, the “Physical Education and Play” course, added to the primary school curriculum by the Ministry of National Education (MEB), is conducted by classroom teachers. This course is structured to provide a fun learning environment through play-based learning and individual, paired, and group activities [13]. Over the four years of primary school, with an average of 17 h per week, this course aims to instil active and healthy living habits, encourage learning through discovery, and promote thinking and problem-solving [14]. To support classroom teachers in the physical education and play curriculum, “Physical Activity Cards” (PACs) are distributed. These cards, intended to enhance students’ physical and motor development, include games that can be played in the classroom or schoolyard and are categorised into yellow (primary school level) and purple (middle school level) groups [13]. This study aims to evaluate the effectiveness of these PACs in improving motor proficiency levels and increasing enjoyment of physical activities among primary school students.

Considering this information, research has also focused on implementing physical education and play courses and using PACs. These studies present various positive and negative views regarding achieving the course objectives [15–18]. Yon and Saraç [19] concluded in their research that classroom teachers’ physical education and play lessons were insufficient in supporting students’ physical activity and fitness levels. In a study examining the functionality of the play and physical activities course and the applicability of traditional children’s games, Celayir [20] highlighted that PACs facilitated the course delivery.

When evaluating the effectiveness and efficiency of lessons, findings indicate that physical education and play lessons could be more efficiently conducted in public schools in Turkey due to a need for more materials and facilities [21]. Some studies report that students are unhappy with physical education and play lessons conducted in classrooms rather than outdoors [22]. Çelik et al. [23] found that primary school students enjoy participating in physical activities and games and want more time allocated to these activities in the programs. Additionally, they discovered a high positive correlation between students’ attitudes toward physical education and play and their attachment to school. It is also noteworthy that few studies investigate the relationship between emotional and psychological factors and motor proficiency. Activities were suitable for students’ physical and mental structure, to be carried out in primary schools, and to gain importance in developing students’ interest, desire, and positive attitudes toward physical education and play lessons.

Given that children in primary schools are under the supervision of their teachers all day, classroom teachers play a crucial role in providing opportunities for students to be more physically active [24]. Canli et al. [25] found a significant relationship between physical parameters and academic achievements in secondary school students. Therefore, it is believed that the effective use of Physical Activity Cards (PACs) in primary schools can contribute to more efficient lessons, healthier physical and motor development, and higher levels of enjoyment in physical activity among young people.

Additionally, the creation of physical activity and play areas within the scope of the study, enabling students to spend their break and free time more efficiently, identifying students with sports talent, and providing applied lessons for classroom teachers to approach physical education and play lessons more positively are considered factors contributing to national sports [26]. This study examines the impact of games implemented with PACs on primary school students’ motor proficiency levels and enjoyment of physical activities in Turkey. The primary objective of this study is to investigate the effects of games implemented with PACs (Physical Activity Cards) on students’ motor proficiency and enjoyment of physical activities. Furthermore, the study explores how these effects change over time and examine the potential relationship between motor proficiency and enjoyment of physical activities. To achieve this, the study employs a comprehensive approach, integrating both experimental and correlational methodologies. The data collected will provide insights into how PAC-based interventions can support students’ physical development and engagement in physical activities, contributing to the broader field of physical education.

## Methods

### Study design

The study employed a single-group pre-test-post-test design, an example of a quasi-experimental quantitative research method. The research represented groups (G), pre-test (T1), intervention (X), and post-test (T2). In this research design, the same variable is measured in participants before and after the intervention. Then, the difference between the pre-test and post-test scores is compared [27].

### Study setting and participants

A state school in a more limited rural area was selected for the study. The purposive sampling method, one of the non-probability-based sampling approaches, was used to determine the study group. To ensure the statistical power and reliability of the study, a sample size calculation was performed using G*Power 3.1.9.7 software. For an independent samples t-test, assuming a medium effect size (Cohen’s d = 0.50), an alpha level of 0.05, and a desired power of 0.80, the minimum required sample size was determined to be 84 participants. Our study included a total of 92 third-grade students, which exceeds this calculated minimum, thus ensuring sufficient statistical power to detect meaningful effects. This sample size is considered adequate for the study’s objectives and the planned statistical analyses. Initially, face-to-face interviews were conducted with classroom teachers in the selected primary school to determine the use of PACs in physical education and play lessons. After the interviews, which 13 classroom teachers voluntarily attended, it was found that 11 teachers never used PACs (84.6%), and two teachers used them partially (15.4%). Considering that one of the teachers who partially used PACs was a second-grade teacher and the other was a fourth-grade teacher and that first-graders might have difficulty answering scale questions, the researchers decided to include all third-grade sections in the study, and all students were invited to participate. Two students who were actively licensed athletes were not included in the study. A total of 92 students ($\:\stackrel{-}{X}$_age_=8.48±0.50), 49 girls ($\:\stackrel{-}{X}$_age_=8.44±0.50) and 43 boys($\:\stackrel{-}{X}$_age_=8.53±0.50), studying in the third grade of a primary school in the Ula district of Mugla province, voluntarily participated in the study.

### Data sources and measurement

Two measurement tools were used to collect data. Firstly, students’ levels of motor proficiency were determined using the Test of Gross Motor Development Second Edition (TGMD-2). Measurements were taken in the schoolyard in the morning and under the supervision of class teachers. Participants were divided into small groups, and two researchers evaluated their performances. Before each measurement, the researchers demonstrated a skill following the TGMD-2 protocol. Students were asked to complete a practice trial before starting the test and to perform two trials for each skill. Once the test began, no corrections were made by the researchers. Scores obtained from the two trials were recorded as Object Control Skill, Locomotor Skill, and Total Motor Proficiency scores in the respective subscales.

Additionally, each student’s performance was recorded on video for later review. Another measurement tool used was the “Enjoyment of Physical Activities” scale. The scale questions were administered face-to-face to students in the classroom.

#### The test of gross motor development second edition (TGMD-2)

The TGMD-2 test, developed by Ulrich, was adapted to Turkish culture by Boz and Aytar [28, 29]. This test includes six items for the “locomotor skills” subtest (run, gallop, hop, leap, horizontal jump, and slide) and five items for the “object control skills” subtest (stationary dribble, catch, kick, overhand throw, and underhand roll). While the original scale had six items for object control skills, “baseball batting” was not included in the adaptation to Turkish culture due to its low correlation with object control (0.19). Each skill in the test consists of three to five performance criteria. When a skill is performed correctly, it earns one point, while incorrect performance results in zero points. Test-retest reliability coefficients ranged from 0.80 to 0.97. The validity study of BKMGT-2 resulted in a chi-square value of 201.07; sd: 43; RMSEA 0.088; with CFI, NFI, and GFI values above 0.93, indicating a high model and data fit. The test was valid and reliable for measuring fundamental movement skills (excluding baseball batting) in Turkish children aged 5–10. Locomotor skills scores range from 0 to 50, while object control skills scores range from 0 to 38. The maximum score obtained from the scale is 88 [29].

Ulrich updated the TGMD-2 test in 2017 to the TGMD-3. In the updated test, the “running hurdle jump” skill was replaced with the “standing long jump” skill in the locomotor skills subtest [30]. Additionally, the object control skills subtest now includes the skill of “volleying a bouncing ball with a racket.” However, a Turkish adaptation of the TGMD-3 test has yet to be made. Therefore, it is deemed appropriate to use the TGMD-2 (adapted to Turkish culture as BKMGT-2) test.

Reliability between the researchers was calculated in the test evaluation, and in cases of disagreement, another expert was consulted. 35% of the images were randomly subjected to reliability checks by the lead researcher and an expert. The first coder reached 96% agreement, while the second came 92%. Overall, inter-rater reliability was 94% for blind double samples, exceeding the required threshold of 90%.

#### Physical activity enjoyment scale (PACES)

The Physical Activity Enjoyment Scale (PACES), developed by Mullen et al. [31], was adapted to Turkish and validated for the Turkish population by Özkurt, Küçükibiş, and Eskiler [32]. This scale consists of 8 items and a single dimension, with a 7-point Likert scale. The maximum score that can be obtained from the scale is 56. The authors stated that the adapted measurement tool can be used in non-commercial scientific studies without permission if the source is cited.

### Procedures

The study was structured around a 10-week intervention period, occurring during the spring months from March to May. This timeframe was chosen to ensure consistency across pre-test and post-test conditions for each class. Before the commencement of the study, parental consent was obtained for each child participating, and detailed information about the research objectives and the voluntary nature of participation was provided face-to-face to potential participants.

**Fig. 1:**
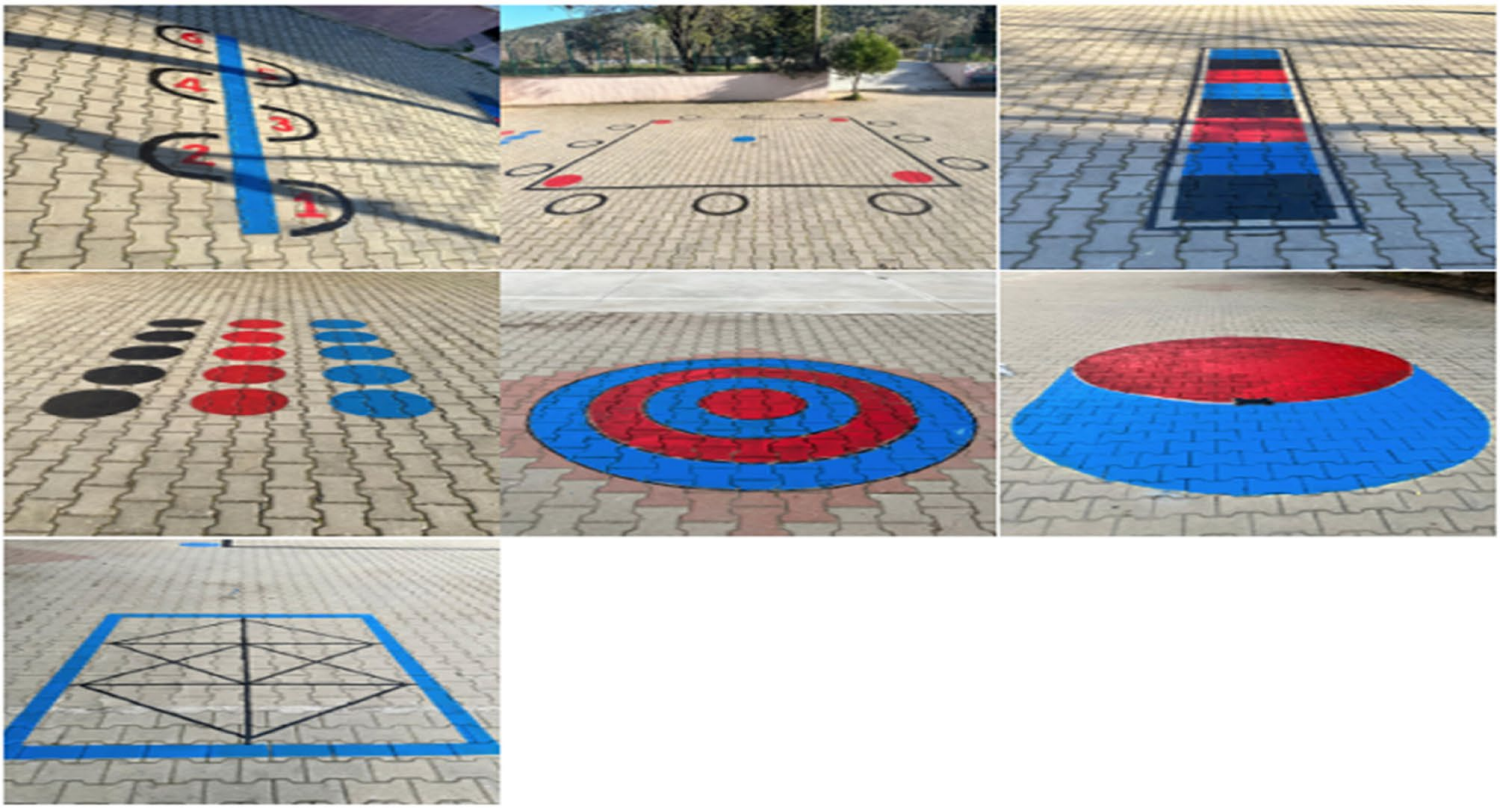
The designed playground based on the PACs

The Physical Activity Cards (PACs) application areas were specifically designed in the schoolyard, and these spaces were prepared with necessary materials for drawing and painting activities, as depicted in Fig. 1. Pilot studies were conducted to familiarise schoolteachers and students with these newly developed areas and the functionality of the PACs.

During the 10-week intervention, students engaged in PAC activities twice a week, with each session lasting 40 min, totalling 160 min of activity per week. The curriculum for these sessions was meticulously planned and updated weekly to maintain engagement and effectiveness. Each session was structured to include a 5-minute warm-up, 30 min of collective or individual games targeting specific skills, and a 5-minute cool-down period.

**Fig. 2:**
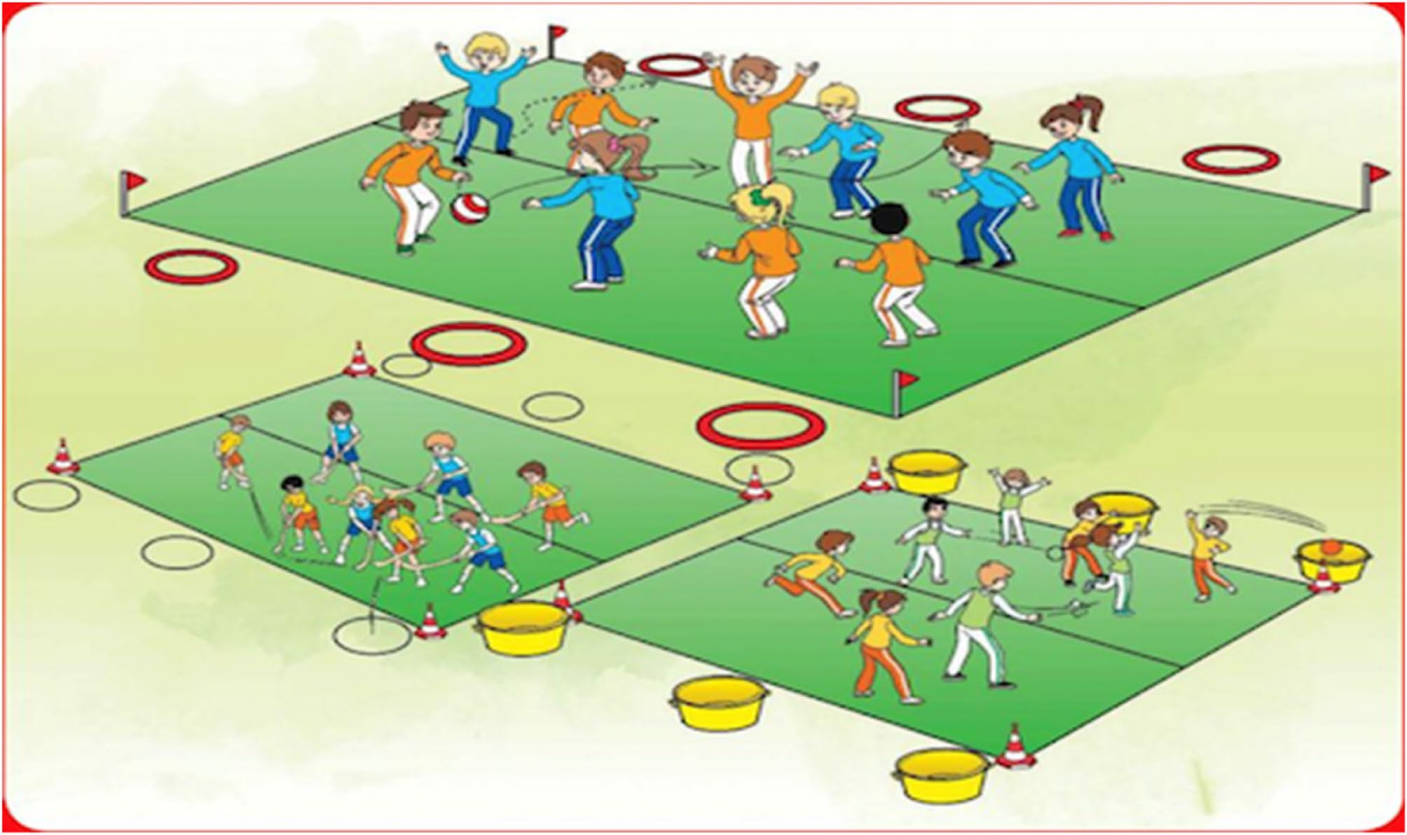
A sample of PACs displaying the playground

Classroom teachers played a pivotal role in facilitating these activities. They were provided with all necessary equipment, such as balls, sandbags, and coloured targets, to support autonomous student engagement in the designated activity areas. Teachers were responsible for guiding the warm-up and cool-down activities, overseeing the games, and ensuring each session adhered to the planned structure. They also monitored student participation, offering the option to opt out of activities or refrain from answering questions at any point, respecting student autonomy and consent Fig. 2.

### Ethical statement

 We asked for parents’ written and signed consent for their children to participate in this study. After obtaining permission, we applied for ethical approval. The study received approval from the Muğla Sıtkı Koçman University Social and Human Sciences Research Ethics Committee (Protocol No: 240064 Decision No: 77), and all ethical guidelines were followed throughout the study. Subsequently, necessary permissions were obtained from the Provincial Directorate of National Education, and face-to-face meetings were held with school administrators and classroom teachers regarding the study plan and the use of PACs.

### Statistical methods

 All data were analysed using the SPSS 26 package program, and a significance level of p<0.05 was set. Before statistical analyses, normality tests were performed to determine whether the data were normally distributed. As the data were found to be normally distributed, independent sample t-tests were used for gender differences, both for scores obtained from the tests and their percentage versions. Paired sample t-tests were used for pre-test-post-test comparisons, and Pearson correlation analysis was conducted to examine the relationship between enjoyment of physical activity and motor skills.

## Results

### Participants and baseline characteristics

This section may be divided by subheadings. It should provide a concise and precise description of the experimental results, their interpretation, and the experimental conclusions that can be drawn.

Table 1 compares the pre-and post-test scores of the Physical Activity Enjoyment Scale (PACES) and the Test of Gross Motor Development (TGMD). This table illustrates significant improvements in motor proficiency and enjoyment after the intervention. Specifically, the PAC program has effectively boosted motor skills and increased enjoyment of physical activities among primary school students, with all changes statistically significant.

**Table 1:** The comparison of the pre/post-test scores of PACES and motor development tests

**Variables**	**n**	**Skewness**	**Kurtosis**	**X̄±ss**	**% ± ss**	**t**	**p**
TGMD GMQ (pre-test)	92	−0.32	−0.35	44.77 ±10.14	50.88 ±11.53	−29.669	.000*
TGMD GMQ (post-test)	92	−0.38	−0.12	55.02 ±10.26	62.52 ±11.66		
TGMD LM (pre-test)	92	−0.20	−0.33	27.48 ±7.22	54.98 ±14.45	−14.243	.000*
TGMD LM (post-test)	92	−0.61	0.09	30.61 ±7.74	61.24 ±15.48		
TGMD OC (pre-test)	92	−0.76	0.59	17.28 ±5.25	45.48 ±13.81	−28.705	.000*
TGMD OC (post-test)	92	−0.94	0.05	26.29 ±5.78	69.19 ±15.22		
PACES (pre-test)	92	−1.08	1.54	37.55 ±8.88	67.06 ±15.86	−18.835	.000*
PACES (post-test)	92	−1.64	3.38	47.35 ±10.14	84.57 ±18.12		

*p<0.05

Table 2 compares the pre-test and post-test scores of TGMD GMQ, TGMD LM, TGMD OC, and PACES between girls and boys. The data reveal gender-specific differences in motor skill enhancements. Girls exhibited more substantial improvements in locomotor skills, while boys showed greater advances in object control skills. Despite these differences in motor skills, the increases in enjoyment levels post-intervention were similar across genders.

**Table 2:** Comparison of TGMD GMQ, TGMD LM, TGMD OC, and PACES pre-test-post-test scores by gender

**Variables**	**Gender**	**n**	**X̄±sd**	**%**±sd	**t**	**p**
TGMD GMQ (pre-test)	Girl	49	46.28**±**10.37	52.60±11.28	1.539	.127
	Boy	43	43.04**±**9.72	48.92±11.05		
TGMD GMQ (post-test)	Girl	49	56.75**±**10.77	64.49±12.25	1.749	.084
	Boy	43	53.04**±**9.38	60.28±10.66		
TGMD LM Pre (pre-test)	Girl	49	29.44**±**6.22	58.89±12.44	2.886	.005^*^
	Boy	43	25.25**±**7.70	50.51±15.40		
TGMD LM (post-test)	Girl	49	33.93**±**5.60	67.88±11.21	4.918	.000^*^
	Boy	43	26.83**±**8.15	53.67±16.30		
TGMD OC (pre-test)	Girl	49	16.83**±**5.77	44.30±15.20	-.868	.388
	Boy	43	17.79**±**4.59	46.82±12.08		
TGMD OC (post-test)	Girl	49	25.14**±**6.38	66.16±16.79	−2.074	.037^*^
	Boy	43	27.60**±**4.75	72.64±12.50		
PACES (pre-test)	Girl	49	38.34**±**8.41	68.47±15.03	.913	.364
	Boy	43	36.65**±**9.40	65.45±16.79		
PACES (post-test)	Girl	49	48.65**±**9.83	86.88±17.56	1.311	.193
	Boy	43	45.87**±**10.41	81.93±18.59		

*p<0.05

Table 3 displays the bivariate correlations between the study variables, including pre-test and post-test scores for the TGMD LM, TGMD OC, TGMD GMQ, and PACES. This table presents the correlations between pre-test and post-test motor proficiency and enjoyment scores. Strong positive correlations are evident, suggesting that improvements in motor skills are closely linked with increased enjoyment. These relationships underscore the effectiveness of the intervention in enhancing interconnected aspects of physical development.

**Table 3:** Bivariate correlations between study variables

**Variables**	**1**	**2**	**3**	**4**	**5**	**6**	**7**
1. TGMD LM (pre-test)	1						
2. TGMD OC (pre-test)	0.30^**^	1					
3. TGMD GMQ (pre-test)	0.87^**^	0.73^**^	1				
4. PACES (pre-test)	0.08	0.16	0.14	1			
5. TGMD LM (post-test)	0.96^**^	0.25^*^	0.81^**^	0.06	1		
6. TGMD OC (post-test)	0.16	0.85^**^	0.55^**^	0.20	0.10	1	
7. TGMD GMQ (post-test)	0.83^**^	0.68^**^	0.94^**^	0.19	0.80^**^	0.65^**^	1
8. PACES (post-test)	0.07	0.13	0.12	0.87^**^	0.08	0.15	0.18

*p< 0.05

**p< 0.01

## Discussion

 This study provides valuable insights into the effects of games related to the PACs) program, implemented over ten weeks, on motor proficiency and enjoyment of physical activities among primary school students. The findings of each research question have been discussed separately.

### Development level of motor proficiency

 The study determined that games related to the PAC program, implemented over ten weeks, positively impacted both dimensions of motor proficiency (Table 3). This result is attributed to the effect of planned games designed to enhance students' movement competence, administered throughout the entire lesson duration by experts in the field. Developed as TOPS cards for use in schools in England and Scotland, the PAC program was introduced as a supplementary material for physical education and physical activities in Turkey in 2012-13 [33]. However, it must still be used at the desired level in physical education and games classes [34]. Additionally, when classroom teachers conduct physical education and games classes in primary schools, problems arise regarding the teaching program and learning outcomes [19, 35–38]. This situation may lead to students having lower physical activity levels and leading more sedentary lifestyles.

Previous studies have indicated that students participate more in lessons conducted with the PAC program, become more interested in games and sports, and show improvements in movement characteristics such as 20 m sprint speed, flexibility, and standing long jump [39–41]. The findings of these studies are consistent with the results of this research. Moreover, systematic review studies in the literature highlight that low physical activity can be a risk factor negatively affecting health, motor proficiency development, and quality of life, and interventions aimed at increasing children's physical activity levels often include activities involving more active games [42, 43]. In our study, active games were effectively implemented. Other studies supporting our findings emphasise children's motor development changes due to intervention programs [44, 45].

Furthermore, researchers observed that the areas created in the schoolyard attracted students' interest, and they played the games learned during the study during recess and free hours. Verstraete et al. [46] emphasise that providing play equipment during recess can increase primary school children's daily physical activity levels. Similarly, Hyndman et al. [47], in their study conducted in two primary schools in Australia, highlight providing opportunities and facilities for physical activities during school recess as an essential strategy for improving children's health-related quality of life. This study's results on motor proficiency are consistent with the general motor proficiency literature and contain similar results [48–51].

### Development level of enjoyment of physical activities

 The study revealed that games related to the PAC program, implemented over ten weeks, positively affected the level of enjoyment of physical activities among primary school students (Table 3). It should be considered that physical, psychological and emotional factors mediate increased participation in physical education and games classes [52, 53]. Sotos-Martínez et al. [54] noted that a gamified intervention increased students' psychological satisfaction and intrinsic motivation while decreasing amotivation. This study supports our findings. Additionally, previous research adopting a holistic perspective, including different age groups, supports our findings [55–57].

Given that primary school students are at an age where school serves as a place for playing with friends and they make sense of their lives through games, this study, which integrates play into education, positively contributed to their enjoyment of physical activities. Mo et al. [58] conducted a systematic review and found that physical game interventions enhanced enjoyment and promoted fun as experienced by children and adolescents. Demirtoz and Alpkaya [59] found that school children most enjoyed games like dodgeball when investigating their levels of enjoyment from physical activities. Burns et al. [60], in a meta-analysis of 220 studies, found that school-based physical activity interventions effectively increased enjoyment of physical activity among children and adolescents. These results support our study.

Furthermore, students' enjoyment of physical activities was positively influenced by their enjoyment of the play areas in the schoolyard, where they played both the games learned during the study and traditional children's games during recess. Hyndman et al. [61] examined students' active play during lunch breaks in simple and low-cost play areas created in schools. They found that the more behaviours children develop, such as physical health, enjoyment of physical activity, and personal play activities, the more likely they are to adopt an active lifestyle.

### Gender differences in motor proficiency and enjoyment of physical activities

 One notable observation from our study is the significant difference in Motor Proficiency between girls and boys in the locomotor and object control dimensions. While girls' pre-test and post-test scores in locomotor skills were significantly higher than boys', boys' post-test scores in object control skills were significantly higher than girls' (Table 2). Skills such as galloping and sliding, which are part of the test content, contributed to girls' higher scores in the locomotor dimension. This is because, in traditional children's games, girls tend to play more hopping games such as hopscotch, skipping rope, and musical, rhythmic movement games than boys [62]. Additionally, boys' higher scores in object control skills can be attributed to their tendency to engage in more competitive ball games whenever they find an opportunity [63]. Demirtoz and Alpkaya [59] found that boys enjoyed ball games like ten passes more than girls when investigating the levels of enjoyment from physical activities among school children. When examining the literature on the gender variable, it is observed that some studies support and do not support the research findings [50, 64, 65]. Barnet [64], in a review study, noted that 42 studies investigating the gender variable found positive relationships between object control skills and Motor Proficiency in boys. Bolger et al. [66] found that boys scored significantly higher than girls in object control skills, while girls scored significantly higher in locomotor skills. These results are consistent with our study findings.

Similarly, boys aged 6–9 scored higher in catching and throwing tasks than girls [48]. When examining another problem in the research, it is observed that there was no significant difference between girls and boys in terms of their levels of enjoyment of physical activities according to the gender variable. Since the participant group is in primary school and still at play age, it can be said that both girls and boys enjoy gamified physical activities. Coulter and Woods [67] stated that boys and girls have similar behaviours and attitudes towards enjoying physical activities and physical education classes. Soares et al. [68], in their study examining the differences between boys and girls in terms of motivation for school sports participation, found that participation for fun and enjoyment was equal in both genders. While these studies support our study's findings, some do not support the findings [69, 70]. Johnson et al. [71] found that boys were more active and enjoyed physical education classes more than girls.

### The relationship between enjoyment of physical activity and motor proficiency

 The study found no significant relationship between the level of enjoyment of physical activities and the dimensions of motor proficiency before and after the intervention (Table 3). Motor Proficiency is proficiency in fundamental movement skills, including locomotor and object control skills, and affects physical activity levels [72, 73]. However, these skills should not be interpreted as the only way for individuals to maintain participation in physical activities throughout their lives. Leisterer et al. [74], in their study investigating the predictors of students' enjoyment of physical education, commented on the satisfaction of basic psychological needs and age level, concluding that higher student age predicts a decrease in enjoyment. Since the sample group in the study consisted of children, they experienced gamified activities aimed at increasing motor proficiency without expecting to feel positive emotions. The literature highlights studies that emphasise the critical role of enjoyment in participation in physical activity, although these do not align with our findings [75, 76]. However, similar studies note that participation in physical activities enhances motor proficiency levels, and as motor proficiency levels improve, participation in physical activities increases, indicating a reciprocal dynamic relationship between them [77–79]. As a result, early interventions aimed at positively developing the relationship between enjoyment of physical activity and motor proficiency among primary school children should be supported, as this relationship may be associated with numerous health benefits. This study involved painting and permanently installing playground areas in an elementary school courtyard, allowing children to play freely in these areas. The research results demonstrated that the games related to the PAC program positively affected students' motor proficiency and enjoyment of physical activities.

## Conclusions

This study has demonstrated the impact of the Physical Activity Cards (PACs) in enhancing motor proficiency and enjoyment of physical activities among primary school students. Throughout the ten-week intervention, significant improvements were observed in both motor skills and the students' attitudes towards physical activities, as evidenced by the data collected through the Test of Gross Motor Development and the Physical Activity Enjoyment Scale.

The PACs, designed to be integrated into the existing curriculum, provided structured and enjoyable physical activities that engaged students and improved their motor skills. This is particularly important in increasing physical activity levels and combating sedentary behaviours among children, which are crucial for physical and mental health outcomes.

Furthermore, the study highlighted the role of PACs in creating a more dynamic and interactive learning environment. Using these cards allowed for a varied approach to physical education, where students could explore different movements and games, thereby enhancing their motor skills and enjoyment simultaneously. Given the positive outcomes associated with using PACs, it is recommended that similar intervention programs be implemented in different educational settings to explore their potential benefits across diverse student populations. Long-term studies could also be conducted to assess the sustained impact of PACs on student development over time.

Future interventions might consider the combined effect of structured play cards and enhanced physical environments to maximise student physical activity levels and motor skill development benefits. Educators and policymakers should consider incorporating such tools into the physical education curriculum to foster an active and engaging learning environment that promotes physical health and enjoyment among students.

### Limitations

 This study, while providing valuable insights into the effectiveness of Physical Activity Cards (PACs) in enhancing motor proficiency and enjoyment of physical activities among primary school students, has several limitations that should be considered when interpreting the results. The study was conducted in only one rural state school, which may limit the generalizability of the findings. Different schools may have varying resources, teacher competencies, and student demographics that could influence the effectiveness of the PACs. The intervention lasted only ten weeks. More than this duration may be needed to observe the long-term effects of the PACs on motor proficiency and enjoyment. Longer-term studies could provide more comprehensive data on the sustainability of the observed benefits. The study focused exclusively on third-grade students. The results may not apply to younger or older children, who could respond differently to the PACs based on their developmental stages. The absence of a control group in the study design limits the ability to attribute improvements solely to the PAC intervention. Future studies could enhance the robustness of the findings by including a control group that does not receive the intervention. Using self-reported measures for assessing enjoyment might introduce bias, as students could overestimate their enjoyment due to social desirability or misunderstanding of the questions. This study did not control factors such as the student’s physical activity levels outside of school, dietary habits, and family support for physical activity. These variables could significantly affect the outcomes and should be considered in future research. The effectiveness of the PACs heavily depended on how the teachers implemented the activities. Variations in teacher enthusiasm, understanding, and adherence to the program could influence the results.

## Data Availability

The data is available at 10.6084/m9.figshare.26138758.
